# Supplementary material for: An Overview of the Policy and Market Landscape of Tobacco Production and Control in Mozambique
Source: Int J Environ Res Public Health. 2021 Jan 5;18(1):343. doi: 10.3390/ijerph18010343 (PMC7795975; doi:10.3390/ijerph18010343)
Supplement: Supplementary file 1 [file ijerph-18-00343-s001.pdf]

Supplementary Materials: Table S1: Ministry of Agriculture and Rural Development[1] (Tobacco Production), Table S2: Ministry of Health (Tobacco Control)

Political Framework (2000- and onwards)

Table S1. Ministry of Agriculture and Rural Development[2] (Tobacco Production)

Policy/ Document	Description	Time	Priority Crops	Stakeholder(s)	Tobacco Promotion?	Promotion Details
1.Regulation on Tobacco Promotion, Production and Marketing	Objective: regulates tobacco production; and contracts between the private sector and the state.	2001	N/A; Tobacco's crop of focus.	N/A	Yes	Regulation for tobacco production.
2.Strategic Plan for Agrarian Sector Development (PEDSA)	Objective: the PEDSA emerges as a guiding framework, harmonizing instrument and mobilizer of synergies to boost agricultural development. This plan:<br>◦ Contains a vision with a short, medium and long-term horizon for the development of agricultural production<br>◦ Aligns the guidelines and practices for agricultural production at national level;<br>◦ Articulates interventions and facilitates access to the resources and means necessary for agricultural production.	2011-2020	Sorghum, Maize, cassava.<br>sweet potatoes and rice (Zambezia and Sofala), Peanuts (Southern provinces w/ exception of Inhambane).<br>-Sesame, tobacco (tete), Sesame and sunflower (Manica). Animal production: Chicken (the whole country), cattle production (Gaza, Inhambane and Tete).	SHFs, Public and Private Institutions	Yes	The production of cash crops (Cotton,canesugar and tobacco) is generally done through a system of concessions in which the State signs contracts for promotion and rural extension, assigning territories to concessionary companies, which in turn provide technical assistance and training to producers, in addition to providing production inputs. (seeds, fertilizers, pesticides, sacks) and, in some cases, investment credit.

[1] Policy Documents/ Legislation relevant to Tobacco are highlighted in yellow.

[2] Policy Documents/ Legislation relevant to Tobacco are highlighted in yellow.

3.Agricultural Sector National Investment Plan (PNISA, in Portuguese)	*PEDSA's operational Plan.*<br>Objective: PNISA reaffirms the vision of the agrarian sector established in PEDSA, of developing "a prosperous, competitive, equitable and sustainable agrarian sector" whose objectives are "to contribute to food security; increase the income and profitability of agricultural producers; and increase market-oriented agrarian production in a fast, competitive and sustainable manner".<br>- PNISA's specific objectives are: (a) To accelerate the production of basic food products (b) to guarantee income for producers, (c) to guarantee access and secure possession of the necessary natural resources, (d) to provide specialized services oriented to the development of the value chain , and (e) boost the development of areas with the greatest agricultural and commercial potential.	2013-2017	Priorities for production are food and cash crops. The priority food crops include: maize, rice, wheat, beans, cassava, tomatoes, potatoes, sweet potatoes and tomatoes; and priority cash crops are cashew, cotton, soy, sesame and tobacco.	SHFs, Public/Private sector.	Yes	Accompanied by increased demand in international markets and the introduction of tobacco production and commercialization in Mozambique, it led to the growth of about 15.5 times the volume of production reaching 72,000 tons from about 130,000 peasant families.<br>-The tobacco support sub-programme aimed to increase production and productivity, improve tobacco classification, conflict management, reforestation in tobacco production areas, implement good agricultural practices (crop diversification for tobacco producers) and monitoring.
4.Agricultural Marketing Operational Plan (POCA, in portuguese)	Objective: Plans have been elaborated at provincial level<br>-Operational Plan for Agricultural Marketing 2018 with a view to guiding its actions in the interconnection of the main actors involved in the value chain of agricultural marketing.	2017-2018	varies by province	SHFs, Public Institutions, Private Sector	Yes (but varies by province, namely Tete,	Part of the main agro-processing industries in these provinces.

	-Therefore, the mapping of the main actors in the agricultural marketing process were carried out by province, and also the mapping of the main agro-processing industries, to ensure the flow of the entire agricultural surplus.<br>-The implementation of the Operational Plan for Agricultural Marketing necessarily involves the implementation of inter-sectoral actions articulated at central, provincial and district levels.				Manica, Zambezia and Niassa)	
5.Agricultural and Livestock Marketing Integral Plan (PICA, in Portuguese)	Objective: PICA is an Instrument for implementing the strategic development plan of the agriculture sector (PEDSA);<br>-Led by the Ministry of Industry and Commerce with the objective of promoting commercialization and stimulate the increase of production and productivity in agriculture.	2013-2020	Maize, rice, wheat, sorghum,beans,peanuts, soy,cashewnuts,tobacco, cotton,sesame,soy and sunflower.	SHFs, Public Institutions, Private Sector	Yes	Mainly Private sector interest -in the case of tobacco (and cotton) if the marketing complies with the system of concessions in which cotton and tobacco companies support producers in inputs and technical assistance on condition that production is purchased by them on an exclusive basis.
6.Agricultural Development Operational Plan (PODA, in Portuguese)	Contributes to food and nutrition security and producer income in a competitive manner, ensuring social and gender equity.<br>OBJECTIVES: (a) ensuring the production of food of plant and animal origin; (b) ensuring food	2015-2019	Maize, rice, beans, horticulture, fruit, chicken production,cattle,cassava,tubercles,sugar,cashews,wheat,soy and cotton.	SHFs, Public Institutions, Private Sector	No	N/A

	and nutritional security; (c) reduce import levels of plant and animal foods; d) Promote the increase in the family income of small producers; (e) promote forest plantations and sustainable management of natural resources (land and water);					
7.National Agricultural Mechanization Program	Objective: to increase the productivity levels by at least 7% as stated in the PEDSA strategy.<br>-Increase ploughed area by using technology packages to increase both production and productivity<br>-Contribute to the increase of income for SHFs through the provision of mechanized services and development of the value chain<br>- Improve the level of preparation of soil and conditions for operations that take place prior to ploughing.	-	Sesame, Maize, bananas, sugar, cashew, cotton, soy and wheat	SHFs, Public Institutions	No	N/A
8. National Agriculture Development Programme (PRO-AGRI)	The PRO-AGRI program was Mozambique's agricultural sector public expenditure program.<br>Objectives: (1) to transform MADER into a modern institution for public sector interventions in support of the agricultural sector.	1999-2004	Food crops: Maize, cassava, rice and beans.;<br>Cash crops: cashews and cotton.	SHFs, public institutions, donors (CIDA)	No	N/A

	(2) To increase agricultural production and productivity in order to improve income and food security for rural households.<br>(3) To protect conservative develop and ensure public access to national resources on sustainable basis.<br>Objectives measured by 8 thematic components (institutional development, research, extension, support for crop production, livestock, forestry and wildlife management, land management, and irrigation)					
Forestry Investment Project - MOZFIP 15	Program to improve land and forest practices and management at the national level.<br><br>Generate impact on (i) Development of family farming and (ii) sustainable management of natural resources, valuing wood and non-wood products at the landscape level.<br>Was implemented in Cabo Delgado and Zambezia.[3]	2017-	N/A	N/A	SHFs, Public Institutions, Private Sector	N/A

[3] Tobacco requires wood in order to dry, however, looking at the geographical areas of implementation MOZ FIP may not be super relevant.

Table S2. Ministry of Health (Tobacco Control)

Policy/Strategic Document/Decree	Time	Stakeholders	Objective	Relation to Tobacco	Stand on Tobacco
Decree for the Consumption and Commercialization of Tobacco, Decree 11/2007	2007	General Public	Legislation to control consumption and commercialization of tobacco	Tobacco control legislation: With the need to regulate the consumption and exposure to tobacco smoke in collective or individual closed places, public or private, through of norms prohibiting tobacco smoke in public places/etc.	Tobacco Control Decree
Strategic Plan for the Health Sector (PESS, in Portuguese)	2014-2019	Government general public, institutions, private sector, academia	National Strategy to lead the production and provision of more and better basic health services, universally accessible, through a decentralized system that privileges partnerships, to maximize the health and well-being of all Mozambicans, which allows them to lead a productive life, towards personal and national development.	Within the PESS, tobacco was mentioned with the 3 health programs under the Ministry of Health (health sector).<br>1. Non Communicable Disease Program, with the objective to reduce the weight of non-communicable disease and mitigate its socio-economic impact. Within this strategy creation of a new legal platform to prevent exposure to risk factors of non-Communicable disease (alcohol, tobacco and healthy diet).<br>2. School Health Program, with the objective of contributing to the establishment of a safe, healthy and favorable school environment for good learning and harmonized development of the student; within this strategy, strengthen coordination mechanisms to support schools in promoting healthy lifestyles. Corners of advice and creation of tobacco-free environments, alcohol and other drugs.	Focused on reducing the exposure to psychoactive substances(with an emphasis on tobacco and alcohol) in order to achieve national health program objectives.

				3. Mental Health Program, with the objective of preventing and reduce morbidity caused by mental disorders and composing, neuropsychiatric diseases and psychosocial distributions, including drug abuse, especially alcohol and Tobacco.<br>In collaboration with DEPROS, information and sensitization activities will be discussed, which raise the level of knowledge of communities about the consequences of the consumption of psychoactive substances, including alcohol and tobacco.<br>*NB: Also included in the indicators for this program in relation to the PESS/Number of annual campaigns of sensabilization and information about the consequences of the consumption of psychoactive substances including alcohol and tobacco.*	
National Strategic Plan for the Prevention and Control of Non-Communicable Disease	2008-2014	Government institutions, general public, private sector, academia	National Strategic Plan for the Prevention and Control of NCDs aims to create a favorable environment to minimize and / or eliminate exposure to risk factors and guarantee access to health care for people who need it.	-Takes into account tobacco is a contributor to NCDs, namely chronic respiratory diseases (asthma), some cancers and cardio vascular disease.<br>*-Breaks down strategic objectives as follows:*<br>*Increase awareness and knowledge about non-communicable diseases and their risk factors in Mozambique.*<br>-Strengthen and integrate capacity building related to these activities in government;<br>Improve coverage, quality and access to prevention and health care services related to non-communicable disease.	Recognizes tobacco as a risk substance for NCDs.

				Strengthen and expand the system of surveillance, investigation, monitoring and evaluation.	
Mental Health Action Plan and Strategy (2007 – 2015)	2007-2015	Government institutions, private sector, general public, academia	• Strengthen the management (planning, organization and financing) of the Health program Mental Health at National Level;<br>• Offer quality psychiatric and mental health services and psychosocial support, comprehensive, integrated and comprehensive at the National Health Service level from primary care from health and community to specialty services;<br>• Implement strategies for promotion and prevention in mental health;<br>• Strengthen mental health information and research systems<br>• Increase public awareness of mental health and reduce stigma and discrimination<br>• Increase community involvement in promoting mental health	Control of abuse of psychoactive substances including alcohol and tobacco. Decree law 11/2007 for tobacco control was underlined as a solution to the objectives.	Recognizes tobacco as a psychoactive substance that can have unfavorable impacts on mental health.

			• Promote and protect the human rights of people with mental illness		
National Plan for Cancer Control	2019-2029	Government institutions, general public,private sector academia	Plan that contains objectives and strategies that will our efforts in six main areas: Prevention, Diagnosis, Treatment, Care Palliative Care, Cancer Registration and Information Systems	-Recognizes tobacco use as one of the contributors to cancer (30% of cases), -Outlines reduction of tobacco use as both an objective and primary intervention to reach policy goals. - Stresses the importance to implementing Decree 11/2007	-Recognizes the need to decrease tobacco consumption as preventative measure for cancer.
